# Time management disposition and academic self-efficacy as predictors of learning well-being among Chinese university students: evidence for a mediation mechanism

**DOI:** 10.3389/fpsyg.2026.1766903

**Published:** 2026-03-25

**Authors:** Huajie Shen, Caixia Bai, Xinzhi Ye, Shi He, Fengwu Zhang, Yushan Yang, Jian Qiu, Jilin Zhang

**Affiliations:** 1 School of Design, Fujian University of Technology, Fuzhou, Fujian, China; 2 School of Education, Fujian Normal University, Fuzhou, Fujian, China; 3 College of Material and Chemical Engineering, Southwest Forestry University, Kunming, Yunnan, China

**Keywords:** academic self-efficacy, learning well-being, mediating effect, post-2000 college students, time management disposition

## Abstract

Learning well-being is an important indicator of students’ positive academic experience, yet the mechanisms linking time management disposition to learning well-being remain insufficiently understood, particularly among Chinese university students born after 2000. This study examined the relationships among time management disposition, academic self-efficacy, and learning well-being, with a particular focus on the mediating role of academic self-efficacy. A questionnaire survey was conducted among 600 Chinese undergraduates from four universities using the College Students’ Learning Well-Being Scale, the Adolescents’ Time Management Disposition Scale, and the Academic Self-Efficacy Questionnaire. Data were analyzed through reliability and validity testing, Pearson correlation analysis, multiple regression analysis, and Bootstrap mediation analysis. The results showed that time management disposition, academic self-efficacy, and learning well-being were all at moderately high levels and were significantly positively correlated. Time efficacy and learning ability self-efficacy emerged as the most stable and strongest positive predictors across multiple dimensions of learning well-being. Mediation analyses further indicated that academic self-efficacy significantly mediated the relationship between time management disposition and learning well-being: time efficacy enhanced academic self-efficacy, which in turn promoted learning well-being, while time value and time monitoring also exerted indirect effects through ability-related and behavior-related efficacy, respectively. These findings suggest that time management disposition contributes to learning well-being both directly and indirectly through academic self-efficacy, extending the literature on self-regulated learning and student well-being by clarifying an efficacy-based mechanism underlying this relationship. Practically, universities may enhance students’ learning well-being by combining time management training with interventions aimed at strengthening academic self-efficacy.

## 1 Introduction

In the Chinese higher education context, the rapid expansion of mass higher education and the deep integration of digital technologies into campus learning have increased students’ demands for autonomy, efficiency, and psychological adjustment. Chinese undergraduates born after 2000 (often referred to domestically as the “post-2000 generation”) have typically grown up with ubiquitous mobile internet access and diversified value systems, which may shape how they regulate learning and evaluate their learning lives. In this study, “born after 2000″ refers to full-time Chinese undergraduates born in or after 2000 who are currently enrolled in undergraduate programs (typically aged 18–24), and the term is used as a cohort label in the Chinese context rather than a strict age-based classification.

Learning well-being, as a key positive psychological indicator of the quality of students’ learning experience, reflects students’ cognitive satisfaction, emotional pleasure, personal growth, and sense of meaning derived from learning (Zandvliet et al., 2019). Understanding how this group attains and sustains learning well-being is thus theoretically meaningful and practically urgent for Chinese universities.

Learning well-being does not exist in isolation; it is profoundly influenced by individuals’ internal cognitive and behavioral dispositions. Among these, time management disposition—defined as a relatively stable psychological and behavioral tendency in the use of time, encompassing one’s cognition of time value, the ability to plan and monitor time, and confidence in managing time effectively (Britton and Tesser, 1991)—has been recognized as an important factor affecting academic achievement and psychological health. Effective time management helps students reduce academic procrastination and stress levels, thereby creating favorable conditions for positive learning experiences (Macan et al., 1990). However, the influence of time management on learning well-being may not be direct; rather, it may operate through other internal psychological mechanisms.

One potential mediating variable is academic self-efficacy, defined as students’ belief in their capability to successfully accomplish academic tasks and achieve learning goals (Locke, 1997). Research indicates that students with higher academic self-efficacy tend to set more challenging goals, show greater persistence in the face of difficulties, and experience lower levels of academic anxiety (Chemers et al., 2001). These adaptive learning behaviors and emotional states are likely to translate directly into greater learning well-being. Moreover, effective time management behaviors—such as planning study schedules appropriately—may enhance students’ sense of control and success in completing tasks, thereby strengthening their academic self-efficacy (Zimmerman et al., 1992). Thus, academic self-efficacy may serve as a bridge linking time management disposition and learning well-being.

Despite growing evidence on time management, self-efficacy, and well-being, two gaps remain (Misra and McKean, 2000). First, prior studies often examine these constructs at an aggregated level, leaving the multidimensional pathways (e.g., time value vs. time efficacy; subjective vs. psychological learning well-being) under-integrated. Second, studies rarely test an integrated, multidimensional model in the specific cultural-educational context of Chinese undergraduates born after 2000.

Therefore, the present study aims to examine the combined effects of time management disposition and academic self-efficacy on learning well-being among “post-2000” college students through a large-scale questionnaire survey. Special attention is given to testing the mediating mechanism of academic self-efficacy between time management disposition and learning well-being. The study proposes a core conceptual framework in which time management disposition not only directly predicts learning well-being but also indirectly enhances it through the promotion of academic self-efficacy. The findings are expected to provide targeted theoretical foundations and practical implications for universities seeking to foster students’ learning well-being through interventions in time management training and self-efficacy development.

## 2 Literature review

### 2.1 Conceptual framework and contemporary significance of learning well-being

Learning well-being (Academic Well-being) is a core construct of positive psychology within the field of education. It transcends traditional criteria of academic achievement by emphasizing students’ positive psychological experiences and personal growth in the learning process (Seligman, 2011). For “post-2000″ college students who have grown up in the digital age, the pursuit of control, meaning, and emotional health in their studies has become increasingly salient amid complex and rapidly changing academic demands (Zhang et al., 2024).

Learning well-being (academic well-being) is rooted in positive psychology and can be approached through two related but distinct traditions. From the subjective well-being perspective, well-being emphasizes cognitive evaluations (e.g., satisfaction) and affective balance (pleasant emotions) in academic life (Dong et al., 2025). From the psychological well-being perspective, well-being highlights optimal functioning, purpose, personal growth, and meaning (Ryff, 1995). Accordingly, learning well-being scales in China often integrate both subjective (e.g., cognitive and affective components) and psychological (e.g., growth and quality components) dimensions (Gao, 2014). This study adopts this integrated perspective to better capture students’ academic thriving.

Recent research has adopted multidimensional perspectives to conceptualize learning well-being. For instance, Zhou and Liu (2025), in their study of heritage language learners, proposed a four-dimensional model encompassing pleasure, connection, meaning, and competence, offering a more nuanced framework for understanding learning well-being. In terms of measurement, Arslan et al. (2022) developed the Subjective Academic Well-being Scale, which operationalizes the construct as a unidimensional structure, providing an efficient tool for assessing students’ positive academic functioning. Collectively, these studies indicate that learning well-being is not a unidimensional affective experience, but rather a comprehensive psychological state integrating cognitive, emotional, and motivational dimensions.

### 2.2 Time management disposition

The concept of time management was first defined by Lakein and Leake (1973) as a structured process involving goal setting, prioritization, and planning tasks to achieve objectives. The construct entered the realm of psychology in the 1960s, initially through research on time attitudes (Calabresi and Cohen, 1968) and individual time experience (Wessman, 1973). In the 1980s, scholars such as Britton and Glynn (1989) systematically proposed the time management behavior theory, which divides time management into three hierarchical levels: the macro level, focusing on goal setting and prioritization; the meso level, concerning task generation and planning; and the micro level, emphasizing task execution.

Based on this framework, Britton and Tesser (1991) developed the Time Management Questionnaire (TMQ), which includes dimensions such as short-term planning, time attitude, and long-term planning. Similarly, Macan et al. (1990) proposed the Time Management Behavior Scale (TMB), encompassing goal setting, planning, perceived control of time, and the tendency toward disorganization. Overall, time management extends beyond the rational allocation and utilization of time; it also integrates individuals’ clear awareness of task goals and their capacity for self-regulation.

Time management has become even more critical in online and blended learning, where students face weaker external structure and higher demands for self-regulation. Recent evidence shows that time management is associated with engagement and adaptive study behaviors in college settings (Fu et al., 2025). Person-centered research on online homework management also highlights time management as a key feature differentiating students’ online learning profiles (Xu, 2025). Reviews of self-regulated learning in blended learning further identify goal setting, monitoring, and time management as recurrent strategies that support learning outcomes.

### 2.3 Academic self-efficacy

Academic self-efficacy originates from Bandura’s social cognitive theory and refers to an individual’s belief in their ability to organize and execute learning activities to achieve specific academic goals (Bandura, 1997). Recent studies have continuously expanded our understanding of how academic self-efficacy develops. A 2025 study on Chinese university students found that future time perspective significantly enhanced academic self-efficacy through the chained mediating effects of personal growth initiative and learning engagement (Zhu et al., 2025). This finding highlights how future-oriented cognitive motivation can be transformed into confidence in one’s abilities through a series of positive psychological and behavioral processes.

Moreover, a meta-analysis integrating data from 23 studies confirmed that self-efficacy serves as a critical mediating variable between intrinsic/extrinsic motivation and academic performance (Yang et al., 2025). This evidence not only reinforces the centrality of self-efficacy in learning processes but also suggests that fostering students’ intrinsic motivation to enhance their self-efficacy may be an effective pathway for improving academic experience and achievement.

### 2.4 Integrative model of time management disposition, academic self-efficacy, and learning well-being

Integrating time management disposition, academic self-efficacy, and learning well-being into a unified model has become a forefront topic in contemporary educational psychology, with emerging empirical evidence supporting this approach.

Both theoretical reasoning and preliminary empirical studies support the role of academic self-efficacy as a key mediating variable. Time management behaviors—such as effective planning and task execution—provide students with direct mastery experiences, which are the most powerful sources of self-efficacy enhancement (Bandura, 1997). When students successfully complete learning tasks through effective time management, they not only achieve their goals but also reinforce their belief in their own competence. This strengthened sense of academic self-efficacy subsequently promotes more positive learning emotions, greater engagement, and deeper satisfaction with the learning process (Zhou and Liu, 2025).

It is noteworthy, however, that the effect of academic self-efficacy may not follow a simple linear pattern. Fokkens-Bruinsma et al. (2021) found that academic self-efficacy plays differentiated roles in alleviating learning anxiety and influencing learning well-being across different student populations. This suggests potential heterogeneity among “post-2000” college students in the pathways through which time management affects learning well-being via self-efficacy.

**Figure 1:**
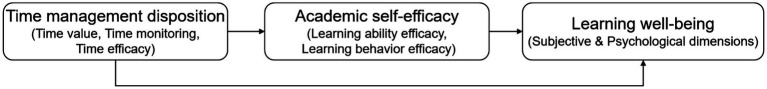
Conceptual model of the study.

In summary, the existing literature clearly underscores the importance of time management disposition and academic self-efficacy in shaping college students’ learning well-being and preliminarily reveals the mediating function of academic self-efficacy within this relationship. However, empirical research specifically focusing on Chinese “post-2000″ college students that integrates multidimensional time management disposition, academic self-efficacy, and multidimensional learning well-being within a single analytical framework remains limited. The present study seeks to fill this gap by testing a mediation model centered on academic self-efficacy, proposing that time management disposition promotes learning well-being both directly and indirectly through the enhancement of academic self-efficacy. Integrating time management disposition, academic self-efficacy, and learning well-being into one framework aligns with self-regulated learning models in which learners use strategies (including time management) to regulate cognition and motivation, and these beliefs and strategies jointly influence well-being outcomes. Figure 1 presents the conceptual model tested in this study.

### 2.5 Research hypotheses

Based on the preceding literature review and the proposed integrative model, the present study formulates the following research hypotheses.

*H1*: Time management disposition is positively associated with learning well-being among “post-2000” college students.

*H2*: Time management disposition is positively associated with academic self-efficacy.

*H3*: Academic self-efficacy is positively associated with learning well-being.

*H4*: Academic self-efficacy mediates the relationship between time management disposition and learning well-being.

## 3 Research methodology

This study employs a combination of questionnaire surveys and quantitative analysis to systematically evaluate the relationships among peer relationships, employment pressure, and learning well-being. The research methodology includes participant recruitment, selection of measurement tools, survey procedures, and statistical analysis. The specific details are as follows.

### 3.1 Participants

Participants were Chinese undergraduates born after 2000 recruited from four universities in China: Fujian University of Technology, Fujian Normal University, Sanming University (all located in Fujian Province), and Southwest Forestry University (located in Kunming, Yunnan Province). A total of 650 questionnaires were distributed and 600 valid responses were collected (valid rate = 92.3%). We used a multi-stage cluster convenience sampling approach: first, four universities with research access were selected; second, intact classes within different grades were invited via the student affairs offices; third, participation was voluntary. To improve representativeness, we aimed to cover multiple grades and majors within each university.

The demographic distribution was as follows:

Grade: Freshman (22.5%), Sophomore (24.5%), Junior (26.5%), Senior (26.5%);

Gender: Male (51.5%), Female (48.5%).

This diverse sampling ensured adequate representativeness across different academic years and personal characteristics.

### 3.2 Measures

To examine the effects of time management disposition and academic self-efficacy on learning well-being among “post-2000” college students, three standardized questionnaires were employed, all of which have demonstrated good reliability and validity in previous research.

(1) College Students’ Learning Well-Being Scale.

The College Students’ Learning Well-Being Questionnaire developed by Gao (2014) was used to assess students’ subjective and psychological well-being in learning. The instrument consists of two subscales—subjective learning well-being and psychological learning well-being—and includes four dimensions: cognitive well-being, affective well-being, quality well-being, and growth well-being. Responses were rated on a 5-point Likert scale ranging from 1 (“strongly disagree”) to 5 (“strongly agree”). The split-half reliability coefficient was 0.971, Cronbach’s *α* was 0.939, indicating good reliability.

(2) Adolescent Time Management Disposition Scale (ATMD).

The Adolescent Time Management Disposition Scale (ATMD) developed by Huang and Zhang (2001) was adopted to measure students’ stable psychological and behavioral tendencies regarding time use. The scale contains 44 items divided into three dimensions: time value, time monitoring, and time efficacy. Each item was rated on a 5-point scale from 1 (“completely inconsistent”) to 5 (“completely consistent”), with higher scores indicating stronger time management disposition. The Cronbach’s *α* coefficient for the scale was 0.973, indicating good reliability.

(3) Academic Self-Efficacy Questionnaire.

Academic self-efficacy was measured using the Academic Self-Efficacy Questionnaire developed by Liang (2000) from Central China Normal University. This instrument consists of 22 items across two dimensions: learning ability self-efficacy and learning behavior self-efficacy. Participants rated each item on a 5-point scale ranging from 1 (“strongly disagree”) to 5 (“strongly agree”), with higher scores reflecting greater perceived academic self-efficacy. The Cronbach’s α coefficient for the questionnaire was 0.916, indicating good reliability.

### 3.3 Procedure

With the assistance of the student affairs departments at the participating universities, the research team distributed the questionnaires both online and offline. Participants voluntarily completed the questionnaires, which took approximately 10–15 min to fill out. Prior to completion, all questionnaires were accompanied by an informed consent form, and no personal identifying information was collected.

### 3.4 Statistical analysis

All data were analyzed using SPSS statistical software. Preliminary analyses included reliability and validity testing to ensure the internal consistency and construct validity of the measurement instruments. Subsequently, Pearson correlation analysis was conducted to examine the relationships among college students’ time management disposition, academic self-efficacy, and learning well-being. Finally, multiple regression analysis was performed to assess the predictive effects of time management disposition and academic self-efficacy on learning well-being.

Because all variables were collected using self-report questionnaires in a single survey, we addressed potential common method bias (CMB) primarily through procedural remedies. Participation was voluntary and anonymous, respondents were informed that there were no right or wrong answers, and the survey was used for academic purposes only. Standardized instructions were provided to reduce evaluation apprehension and socially desirable responding. These steps were intended to minimize the likelihood that a single measurement method systematically inflated the observed relationships among constructs.

## 4 Research results

### 4.1 Descriptive statistics

Descriptive statistics for each dimension of time management disposition, academic self-efficacy, and learning well-being among “post-2000” college students are presented in Table 1. All variables had mean values above the midpoint of the scale (3), indicating an overall moderately high level. Specifically, learning ability efficacy (M = 3.85, SD = 0.79) and learning behavior efficacy (M = 3.90, SD = 0.82) were higher than other variables, suggesting that participants evaluated themselves more positively in learning confidence and behavioral management. One-sample t-tests using the midpoint value (3) as a reference showed that all variables were significantly above the midpoint (min t = 18.71, max t = 27.69, all *p* < 0.001). The “Range (Scale)” column in Table 1 refers to the theoretical range (1–5) rather than the observed sample extremes.

**Table 1:** Descriptive statistics for time management disposition, academic self-efficacy, and learning well-being.

Variable	Mean (M)	SD	95% CI	Range (Scale)
Subjective learning well-being	3.78	0.69	[3.72, 3.84]	1–5
Cognitive well-being	3.82	0.75	[3.76, 3.88]	1–5
Affective well-being	3.75	0.71	[3.69, 3.81]	1–5
Psychological learning well-being	3.78	0.77	[3.72, 3.84]	1–5
Growth well-being	3.76	0.78	[3.70, 3.82]	1–5
Quality well-being	3.80	0.76	[3.74, 3.86]	1–5
Time value	3.70	0.76	[3.64, 3.76]	1–5
Time monitoring	3.55	0.72	[3.49, 3.61]	1–5
Time efficacy	3.60	0.74	[3.54, 3.66]	1–5
Learning ability efficacy	3.85	0.79	[3.79, 3.91]	1–5
Learning behavior efficacy	3.90	0.82	[3.83, 3.97]	1–5

### 4.2 Correlation analysis

Pearson correlation analyses (two-tailed, *N* = 600) were conducted to examine the linear relationships among the main variables. Results are presented in Table 2. Overall, the three dimensions of time management disposition (time value, time monitoring, and time efficacy) were significantly and positively correlated with all dimensions of learning well-being (subjective learning well-being, cognitive well-being, affective well-being, psychological learning well-being, growth well-being, and quality well-being). Similarly, both dimensions of academic self-efficacy (learning ability efficacy and learning behavior efficacy) were positively correlated with learning well-being (all *p* < 0.001). Correlation coefficients ranged from *r* = 0.55 to 0.78, with particularly strong associations between learning ability efficacy and affective well-being (*r* = 0.78), and between time efficacy and psychological learning well-being (*r* = 0.73). As shown in the correlation analysis, several constructs were strongly related to learning well-being (with correlations reaching *r* = 0.78). We therefore treated potential multicollinearity as a consideration when interpreting the regression models. Specifically, rather than relying on a single coefficient in isolation, we evaluated the robustness of findings by examining whether the direction and significance of key predictors remained consistent across (a) different well-being dimensions and (b) alternative model specifications used in the study (e.g., regressions and the bootstrap mediation models). Across analyses, time efficacy and academic self-efficacy displayed stable positive effects, supporting the substantive conclusions regarding the hypothesized mechanism. Nonetheless, future research is encouraged to report formal multicollinearity diagnostics (e.g., tolerance/VIF) alongside regression results to further strengthen statistical inference.

**Table 2:** Zero-order correlations among time management disposition, academic self-efficacy, and learning well-being among post-2000 college students.

Learning well-being dimension	Time value	Time monitoring	Time efficacy	Learning ability efficacy	Learning behavior efficacy
Subjective learning well-being	0.58***	0.64***	0.72***	0.77***	0.71***
Cognitive well-being	0.56***	0.61***	0.70***	0.75***	0.69***
Affective well-being	0.59***	0.65***	0.71***	0.78***	0.72***
Psychological learning well-being	0.62***	0.66***	0.73***	0.74***	0.70***
Growth well-being	0.55***	0.60***	0.68***	0.69***	0.66***
Quality well-being	0.60***	0.63***	0.70***	0.73***	0.68***

*p* < 0.001 indicates a statistically significant correlation.

### 4.3 Regression analysis

A series of multiple regression models were constructed by simultaneously entering the five predictor variables—time value, time monitoring, time efficacy, learning ability efficacy, and learning behavior efficacy. Results are presented in Table 3. All six models for the dependent variables were statistically significant [*R*^2^ = 0.31–0.36, adjusted *R*^2^ = 0.30–0.35, overall *F*(5, 594) = 53.37–66.83, *p* < 0.001].

**Table 3:** Multiple regression analysis of time management disposition and academic self-efficacy on learning well-being among post-2000 college students.

Learning well-being dimension	Predictor	*β*	*SE*	*t*	*p*	*R* ^2^	Adjusted *R*^2^	F (5,594)
Subjective learning well-being	Time value	0.28	0.07	4.00	<0.001	0.34	0.33	61.20
	Time monitoring	0.24	0.08	3.00	0.003			
	Time efficacy	0.32	0.06	5.33	<0.001			
	Learning ability efficacy	0.34	0.06	5.67	<0.001			
	Learning behavior efficacy	0.27	0.07	3.86	<0.001			
Cognitive well-being	Time value	0.22	0.08	2.78	0.006	0.31	0.30	53.37
	Time monitoring	0.26	0.07	3.71	<0.001			
	Time efficacy	0.30	0.07	4.29	<0.001			
	Learning ability efficacy	0.32	0.07	4.57	<0.001			
	Learning behavior efficacy	0.29	0.06	4.83	<0.001			
Affective well-being	Time value	0.25	0.06	4.17	<0.001	0.36	0.35	66.83
	Time monitoring	0.28	0.07	4	<0.001			
	Time efficacy	0.33	0.05	6.60	<0.001			
	Learning ability efficacy	0.33	0.05	6.60	<0.001			
	Learning behavior efficacy	0.28	0.06	4.67	<0.001			
Psychological learning well-being	Time value	0.20	0.07	2.86	0.004	0.32	0.31	55.91
	Time monitoring	0.21	0.08	2.63	0.009			
	Time efficacy	0.29	0.06	4.83	<0.001			
	Learning ability efficacy	0.31	0.06	5.17	<0.001			
	Learning behavior efficacy	0.25	0.07	3.57	<0.001			
Growth well-being	Time value	0.23	0.06	3.83	<0.001	0.33	0.32	58.51
	Time monitoring	0.22	0.07	3.14	0.002			
	Time efficacy	0.30	0.05	6.00	<0.001			
	Learning ability efficacy	0.29	0.06	4.83	<0.001			
	Learning behavior efficacy	0.27	0.06	4.50	<0.001			
Quality well-being	Time value	0.26	0.07	3.71	<0.001	0.35	0.34	63.97
	Time monitoring	0.24	0.07	3.43	0.001			
	Time efficacy	0.31	0.06	5.17	<0.001			
	Learning ability efficacy	0.32	0.06	5.33	<0.001			
	Learning behavior efficacy	0.28	0.06	4.67	<0.001			

Among the individual predictors, time efficacy (*β* ≈ 0.29–0.33) and learning ability efficacy (*β* ≈ 0.29–0.34) emerged as the most stable and strongest positive predictors across dimensions of learning well-being. Other predictors (time value, time monitoring, and learning behavior efficacy) also exerted significant positive effects, though of smaller magnitudes.

Specifically, time efficacy showed the highest standardized coefficients for affective well-being (*β* = 0.33, *t* = 6.60, *p* < 0.001) and growth well-being (*β* = 0.30, *t* = 6.00, *p* < 0.001). In contrast, learning ability efficacy had particularly strong effects on subjective learning well-being, cognitive well-being, and quality well-being (*β* = 0.34, 0.32, and 0.32, respectively; all *p* < 0.001). Although time value and time monitoring exhibited relatively smaller effects, they remained statistically significant across multiple dimensions (e.g., *β* = 0.28, *t* = 4.00, *p* < 0.001; *β* = 0.24, *t* = 3.00, *p* = 0.003).

These findings indicate that, controlling for other factors, improvements in time efficacy and learning ability efficacy are significantly associated with higher levels of multiple dimensions of learning well-being among college students. The study hypotheses (H1–H4) are formulated at the construct level. Because time management disposition, academic self-efficacy, and learning well-being are modeled as multidimensional constructs, the regression results are reported across the corresponding dimensions. Therefore, Table 3 presents coefficients from a set of regression models examining the same theoretical relations across different well-being dimensions and predictor dimensions, rather than representing additional independent hypotheses.

### 4.4 Mediation analysis

To explore the mediating role of academic self-efficacy in the relationship between time management disposition and learning well-being, a parallel mediation model was constructed incorporating time value, time monitoring, time efficacy, learning ability efficacy, and learning behavior efficacy as variables. The mediation effects were tested using the Bootstrap method (5,000 samples, bias-corrected 95% confidence intervals; standardized *β*, *N* = 600). Significant effects were determined by confidence intervals that did not include zero.

For cognitive well-being, Table 4 shows that all three indirect pathways through academic self-efficacy were significant:

**Table 4:** Key mediation and direct effects of academic self-efficacy.

Effect type	Pathway	Effect (*β*)	95% CI	Significance
Indirect effects (DV: Cognitive well-being)	Time value → Learning ability efficacy → Cognitive well-being	0.13	[0.07, 0.20]	Significant
	Time monitoring → Learning behavior efficacy → Cognitive well-being	0.09	[0.04, 0.15]	Significant
	Time efficacy → Academic self-efficacy → Cognitive well-being	0.18	[0.10, 0.26]	Significant
Direct effects (DV: Growth well-being)	Learning ability efficacy → Growth well-being	0.20	[0.12, 0.28]	Significant
	Learning behavior efficacy → Growth well-being	0.18	[0.10, 0.26]	Significant

The indirect effect of time value via learning ability efficacy was *β* = 0.13, 95% CI [0.07, 0.20]; The indirect effect of time monitoring via learning behavior efficacy was *β* = 0.09, 95% CI [0.04, 0.15]; The total indirect effect of time efficacy through overall academic self-efficacy was *β* = 0.18, 95% CI [0.10, 0.26].

These findings indicate that higher time value and time monitoring contribute to improved cognitive well-being indirectly by enhancing students’ learning ability efficacy and learning behavior efficacy, respectively. Meanwhile, time efficacy exerts an overall mediating influence through the comprehensive academic self-efficacy system.

For growth well-being, two significant direct effects of academic self-efficacy were observed:

Learning ability efficacy → growth well-being: *β* = 0.20, 95% CI [0.12, 0.28];

Learning behavior efficacy → growth well-being: *β* = 0.18, 95% CI [0.10, 0.26].

Even after controlling for the three time management dimensions, academic self-efficacy remained significantly and positively associated with growth-oriented learning well-being, demonstrating its unique contribution to students’ developmental well-being.

In summary, the results confirm the pathway “time management disposition → (academic self-efficacy) → learning well-being.” Academic self-efficacy serves as a significant mediator for cognitive well-being and as a direct predictor of growth well-being. This pattern suggests that universities seeking to enhance students’ learning well-being should focus on cultivating both time efficacy and academic self-efficacy, thereby establishing an effective linkage from time management to learning well-being.

## 5 Discussion

This study investigated the effects and mechanisms of time management disposition and academic self-efficacy on learning well-being among “post-2000” college students. Overall, the results showed that the sample scored moderately high on all major variables (Table 1), with medium-to-large correlations among them (*r* = 0.55–0.78; Table 2). Multiple regression analyses revealed that the models explained between 31 and 36% of the variance in learning well-being (Table 3). At the mechanism level, the mediation results (Table 4) demonstrated that, for cognitive well-being, time value, time monitoring, and time efficacy all exerted significant indirect effects through academic self-efficacy, while for growth well-being, academic self-efficacy showed significant direct effects. Together, these findings support the pathway “time management disposition → (academic self-efficacy) → learning well-being,” while also suggesting differential mechanisms across the dimensions of learning well-being.

### 5.1 The influence of time management disposition on learning well-being

Consistent with Hypothesis 1, the results indicate that time management disposition is a significant and stable positive predictor of learning well-being. Regression analyses indicated that time efficacy emerged as the most stable and robust positive predictor across all dimensions of learning well-being (*β* ≈ 0.29–0.33), whereas time value and time monitoring also showed smaller but significant positive effects (Table 3). This pattern suggests that, compared with merely “valuing time” or “monitoring schedules,” individuals’ confidence in their ability to manage time effectively more consistently translates into positive learning experiences and emotional evaluations.

Combined with the magnitude of correlations (Table 2), the results confirm that time management is not only broadly associated with learning well-being but also makes an independent contribution when other variables are controlled. This aligns with prior findings in educational and psychological research (e.g., Macan et al., 1990; Britton and Tesser, 1991), which emphasize that practicable and sustainable time management efficacy is a crucial bridge linking learning behaviors to positive psychological outcomes.

### 5.2 The influence of academic self-efficacy on learning well-being

These findings provide support for Hypothesis 3, demonstrating that academic self-efficacy plays a crucial role in promoting students’ learning well-being. In addition, the positive association between time management disposition and academic self-efficacy lends further support to Hypothesis 2. Both learning ability efficacy and learning behavior efficacy were found to be significant positive predictors across multiple dimensions of learning well-being (Table 3). Notably, learning ability efficacy showed stronger effects on subjective, cognitive, and quality well-being (*β* ≈ 0.32–0.34). This indicates that students’ positive beliefs about their learning abilities and strategic competence are not only associated with a higher quality of learning experiences but may also contribute to broader well-being gains through increased task engagement, strategic learning behavior, and emotional regulation.

Consistent with the social-cognitive perspective (Bandura, 1997), efficacy beliefs serve as core psychological resources that drive goal persistence, emotion management, and self-regulatory behavior. Students with stronger efficacy beliefs are therefore more likely to experience satisfaction, meaning, and emotional balance in the learning process—key components of learning well-being.

### 5.3 Mediating role and dimensional differences of academic self-efficacy

Overall, the mediation analyses support Hypothesis 4, confirming that academic self-efficacy serves as a key psychological mechanism linking time management disposition to learning well-being. As shown in the key mediation results (Table 4), all three indirect paths from time value, time monitoring, and time efficacy to cognitive well-being via academic self-efficacy were significant (*β* = 0.09–0.18, BCa 95% CI excluding zero). This indicates that time management disposition contributes to cognitive-oriented learning well-being through enhanced academic self-efficacy. Specifically, time value exerted its influence primarily through learning ability efficacy, time monitoring through learning behavior efficacy, and time efficacy through the overall academic self-efficacy system.

In contrast, for growth well-being, academic self-efficacy demonstrated two significant direct effects: learning ability efficacy (*β* = 0.20, 95% CI [0.12, 0.28]) and learning behavior efficacy (*β* = 0.18, 95% CI [0.10, 0.26]). These findings suggest that academic self-efficacy independently contributes to students’ developmental well-being, even after accounting for time management variables.

Overall, academic self-efficacy plays differentiated roles across the dimensions of learning well-being: it functions as a mediating mechanism for cognitive well-being, while serving as a direct contributor to growth-oriented well-being. This nuanced pattern highlights the multifaceted nature of self-efficacy in promoting both the cognitive and developmental aspects of students’ learning happiness.

Taken together, the findings provide empirical support for the proposed hypotheses, demonstrating that time management disposition enhances learning well-being both directly and indirectly through academic self-efficacy.

### 5.4 Cross-cultural implications

Although the proposed mechanism (time management disposition → academic self-efficacy → learning well-being) was supported in this sample of Chinese undergraduates, the strength and expression of this pathway may vary across cultural and educational contexts. In Chinese higher education, relatively high achievement expectations and normative emphasis on diligence and efficiency may strengthen the motivational value of perceived time control, thereby amplifying its contribution to competence beliefs and learning well-being. In contrast, in educational systems that are less structured or place greater emphasis on autonomy rather than efficiency norms, time-related self-regulation may relate to learning well-being more through autonomy, interest, or other motivational routes, and the central role of time efficacy may depend on learners’ goal orientations and institutional demands. While this study highlights academic self-efficacy as a key mediator, time management may also relate to learning well-being through parallel pathways such as stress reduction and engagement. In addition, some constructs are conceptually proximal; future work may further test boundary conditions across learning contexts.

### 5.5 Theoretical and practical implications

First, this study links time-related self-regulatory resources to learning well-being by clarifying an efficacy-based mechanism, thereby extending research that typically treats time management as a behavioral skill without specifying how it translates into well-being outcomes. Second, by modeling constructs multidimensionally, the findings suggest that not all time-management components function equivalently: perceived time efficacy appears more consistently related to well-being than time value or time monitoring alone, which helps refine theoretical accounts of self-regulation quality versus mere behavioral compliance. Third, the dimension-level results imply that different facets of academic self-efficacy (learning ability vs. learning behavior efficacy) may contribute differently across well-being dimensions, providing a more nuanced view of how competence beliefs support students’ cognitive, affective, and developmental experiences in learning.

For educational practice, interventions should prioritize strengthening students’ time efficacy (confidence in managing time effectively) rather than focusing only on scheduling or monitoring techniques. Universities may combine time-management training with self-efficacy-enhancing strategies such as mastery-oriented task scaffolding, feedback that emphasizes progress and competence, and peer modeling of effective study strategies. In addition, student support services could differentiate support by well-being domains (e.g., cognitive/subjective vs. growth/quality well-being) and tailor programs that jointly address time-related control, competence beliefs, and engagement in learning activities.

## 6 Conclusion

This study conducted an empirical investigation among Chinese “post-2000” undergraduates from multiple universities, examining how time management disposition (time value, time monitoring, time efficacy) and academic self-efficacy (learning ability efficacy, learning behavior efficacy) are associated with multidimensional learning well-being (subjective, cognitive, affective, psychological, growth, and quality well-being). Using validated measurement scales and a combination of correlation analysis, regression models, and bootstrap-based mediation testing, the study provides evidence on both the overall relationships and the underlying mechanism across well-being dimensions.

The findings can be summarized as follows. First, participants generally reported a moderately high level of time management disposition, academic self-efficacy, and learning well-being, and the three constructs showed consistent positive associations across all well-being dimensions. Second, when the multidimensional predictors were considered simultaneously, time efficacy and learning ability efficacy emerged as the most robust predictors across multiple dimensions of learning well-being, while time value, time monitoring, and learning behavior efficacy also contributed in a weaker but meaningful manner. Third, the mediation tests indicate that academic self-efficacy plays a key explanatory role in linking time management disposition to learning well-being, particularly for the cognitive and developmental facets of well-being. Specifically, students’ perceived effectiveness in managing time (time efficacy) appears to strengthen academic self-efficacy, which in turn supports better learning well-being; meanwhile, time value and time monitoring show more differentiated pathways through ability- and behavior-related efficacy, respectively.

Overall, the results support the proposed model that time management disposition promotes learning well-being through academic self-efficacy, while also highlighting that the effects operate differently across dimensions of learning well-being. These findings underscore the importance of focusing not only on time management behaviors, but also on students’ perceived competence and confidence in managing their learning process, which may offer practical leverage points for universities seeking to enhance students’ learning well-being.

Despite its contributions, this study has several limitations. First, the cross-sectional design limits causal inference; future studies are encouraged to adopt longitudinal tracking or experimental/intervention-based designs to validate the proposed relationships. Second, all measures were collected via self-report at a single time point, which may introduce common method variance and inflate observed associations. Future research could address this concern by incorporating multi-source data (e.g., learning logs or behavioral analytics), using time-separated measurement designs, and applying additional statistical diagnostics (e.g., Harman’s single-factor test, marker variables, or latent method factor approaches). In addition, given the relatively strong associations observed among several constructs, formal multicollinearity diagnostics (e.g., tolerance/VIF) were not reported in the present manuscript and should be included in future analyses to further evaluate coefficient stability. Where appropriate, SEM-based approaches may also be considered to model the measurement and structural relations simultaneously and to provide additional evidence for the distinctiveness of closely related predictors. Third, the sample was primarily drawn from universities in specific regions of China, which may constrain external validity. Future work could expand sampling across more regions and institutional types, and integrate additional psychological and contextual variables (e.g., emotion regulation, social support, and learning engagement) to refine the model’s explanatory and predictive power and to enhance generalizability across diverse student populations.

## Data Availability

The original contributions presented in the study are included in the article/supplementary material, further inquiries can be directed to the corresponding author.
